# Identification of novel mutations in *HEXA* gene in children affected with Tay-Sachs disease from India

**DOI:** 10.1186/1755-8166-7-S1-P53

**Published:** 2014-01-21

**Authors:** Mehul Mistri, Chaitanya Datar, Frenny Sheth, Sarita Gupta, Jayesh Sheth

**Affiliations:** 1 FRIGE’s Institute of Human genetics, Ahmedabad, Gujarat, India; 2 Sahyadri Medical Genetics and tissue engineering facility (SMGTEF), Pune, Maharashtra, India; 3 The M. S. University, Vadodara, Gujarat, India

## Background

Tay-Sachs disease (TSD) is an autosomal recessive storage disorder due to impaired activity of the lysosomal enzyme β-Hexosaminidase-A (EC 3.2.1.52) due to the mutation in *HEXA* gene. As per HGMD database, 134 mutations have been reported from different ethnic groups, while in India only few mutations have been reported till date. Here we reported three new novel mutations in *HEXA* gene causing TSD in children from Maharashtra. The objective of the investigation was to determine the disease causing mutations in *HEXA* gene in children affected with Tay-Sachs disease confirmed by deficient enzyme activity of β-Hexosaminidase-A.

## Materials and Methods

Seven children in the age range of 1 month to 1.5 years were enrolled in this study. Enzyme study was carried out using 4-MU substrate (MUGS) specific for β-Hexosaminidase-A. The exons and exon-intron boundaries of *HEXA* gene were bidirectionally sequenced using automated sequencer. In silico analysis was carried out using SIFT, Polyphen2 and Mutation T@ster softwares. Written consent was obtained from guardian of the study subjects.

## Results

Overall, we have identified 8 mutations in seven unrelated families, three of which are novel, including combined heterozygous missense mutations c.524A>C (p.D175A) and c.805G>C (p.G269R) was observed in one case and one homozygous nonsense mutations c.1528C>T (p.R510X) in one case. A previously known missence mutations c.532C>T (R178C), c.964G>T (p.D322Y), c.1385A>T (p.E462V), 4bp insertion c.1277_1278insTATC (p.Y427Ifs5) and splice site mutations c.459+5 G>A were observed in 5 children. In silico analysis further confirmed the pathogenic effect of the novel mutations occurred at highly evolutionarily conserved and functionally active domain residues in the protein leading to conformational changes or mRNA producing truncated protein resulting in the diminish or absent activity of the protein.

## Conclusion

Mutations responsible for TSD in Indian population are unique. We have found 3/8 (37.5%) novel mutations [D175A, G269R and R510X] in present study along with 5/8 (62.5 %) previously reported mutations [E462V, D322Y, R178C, c.1277_1278insTATC (p.Y427IfsX5) and c.459+5 G>A]. This study further confirms that nearly 37.5 % of TSD children harbor novel mutations in India and 62.5 % have common or earlier reported mutations. Overall study provides the new insights into the molecular basis of the disease that can be utilized for the molecular screening of TSD.

